# Scaffold-based delivery of mesenchymal stromal cells to diabetic wounds

**DOI:** 10.1186/s13287-022-03115-4

**Published:** 2022-08-20

**Authors:** Shanshan Du, Dimitrios I. Zeugolis, Timothy O’Brien

**Affiliations:** 1 Regenerative Medicine Institute (REMEDI), Biomedical Sciences Building, National University of Ireland Galway (NUI Galway), Galway, Ireland; 2 Science Foundation Ireland (SFI) Centre for Research in Medical Devices (CÚRAM), Biomedical Sciences Building, National University of Ireland Galway (NUI Galway), Galway, Ireland; 3 Regenerative, Modular and Developmental Engineering Laboratory (REMODEL), Biomedical Sciences Building, National University of Ireland Galway (NUI Galway), Galway, Ireland; 4 Regenerative, Modular and Developmental Engineering Laboratory (REMODEL), Charles Institute of Dermatology, Conway Institute of Biomolecular and Biomedical Research and School of Mechanical and Materials Engineering, University College Dublin (UCD), Dublin, Ireland

**Keywords:** Diabetic wound healing, Mesenchymal stromal cells, Biomaterials

## Abstract

Foot ulceration is a major complication of diabetes mellitus, which results in significant human suffering and a major burden on healthcare systems. The cause of impaired wound healing in diabetic patients is multifactorial with contributions from hyperglycaemia, impaired vascularization and neuropathy. Patients with non-healing diabetic ulcers may require amputation, creating an urgent need for new reparative treatments. Delivery of stem cells may be a promising approach to enhance wound healing because of their paracrine properties, including the secretion of angiogenic, immunomodulatory and anti-inflammatory factors. While a number of different cell types have been studied, the therapeutic use of mesenchymal stromal cells (MSCs) has been widely reported to improve delayed wound healing. However, topical administration of MSCs via direct injection has several disadvantages, including low cell viability and poor cell localization at the wound bed. To this end, various biomaterial conformations have emerged as MSC delivery vehicles to enhance cell viability and persistence at the site of implantation. This paper discusses biomaterial-based MSCs therapies in diabetic wound healing and highlights the low conversion rate to clinical trials and commercially available therapeutic products.

## Introduction

Diabetic foot ulcers are the main cause of amputation in patients with diabetes mellitus, resulting in high healthcare costs, reduced quality of life and increased mortality. The 10th edition of the International Diabetes Federation Atlas has reported that the number of patients suffering from diabetes mellitus in 2021 was 536.6 million, and there will be approximately 783.2 million adults with diabetes by 2045 [1]. The global health expenditure on diabetes mellitus is estimated to reach US$ 1,054 billion by 2045, increasing 9.1% from US$ 966 billion in 2021 [2]. The global epidemiology of diabetic foot ulcers has been reported to have a prevalence of 6.3%, which was higher in males than females, and in patients with type 2 diabetes mellitus than in those with type 1 (6.4% vs 5.5%) [3]. From a global perspective, the prevalence of diabetic foot ulcers in North America, Asia, Europe, Africa and Oceania was 13.0%, 5.5%, 5.1%, 7.2% and 3.0%, respectively [3]. It is estimated that approximately 25% of patients with diabetes mellitus will develop a foot ulcer during their lifetime [4], and around 14–24% of patients with foot ulcers will ultimately require an amputation [5]. In the UK, the number of patients with chronic wounds was estimated to be 2.1 million, increasing at the rate of 12% annually with annual health care expenditure of approximately £5 billion [6]. In the United States, approximately 6.5 million people experience diabetic foot ulcers with the cost for wound care management in the range of US$ 28.1 to US$ 96.8 billion [7, 8]. Therefore, diabetes mellitus and the complication of foot ulceration have major human, societal and economic costs.

To date, numerous approaches have been developed for chronic wound management and treatment, such as gene therapy, growth factor therapy, stem cell therapy, and use of biomaterials. Current treatment methods for diabetic wound healing are not always effective [9, 10]. The basic care of neuropathic foot ulcers consists of wound debridement and off weight bearing, while ischemic ulcers require revascularization. It has been well-established in the literature that MSCs secrete growth factors, cytokines, and chemokines which may contribute to the therapeutic potential in the context of diabetic wound healing [11–13]. In addition, MSCs impact each phase of the wound healing process via modulation of immune responses, and promotion of angiogenesis and tissue remodelling [13, 14].

Current cell-based treatments are mainly focused on cell injections either delivered systemically or intradermally. MSCs administrated via the systemic route have shown that the majority of cells entrap in the lung, and only a small portion of cells travel to the wound site [15], whereas intradermally injecting MSCs into the wound edges significantly improved the wound healing process [16]. However, the therapeutic effect of MSCs can still be compromised by poor cell localisation and impaired cell viability at the site of injury [17]. To overcome these problems, the use of scaffolds has been advocated as a means to increase cell viability and retention at the wound bed and provide a three-dimensional structure for cell migration, proliferation and differentiation [18]. Herein, we summarize the underlying mechanism of MSC mediated diabetic wound healing, along with significant advances and shortfalls of biomaterial-based MSC therapies in diabetic wound healing in preclinical and clinical settings.

## MSC mediated diabetic wound healing

Normal wound healing progresses through four overlapping phases: haemostasis, inflammation, proliferation, and remodelling. Diabetic wounds are characterised by a delayed inflammatory phase, and therefore take a long time to heal [19]. In most patients, the underlying etiology of diabetic wounds is mainly due to a combination of factors such as peripheral neuropathy, peripheral artery disease and impaired immune response [20]. Neuropathy results in impaired sensory, motor, and autonomic nerves leading to inability to detect external stimuli such as pressure, heat and the creation of wounds [21, 22]. Peripheral artery disease results in ischemia and microcirculatory dysfunction, leading to a decrease in local angiogenesis [23]. Some patients also show a reduced immune response to infections which inhibits wound healing [24]. Collectively, multiple factors contribute to the prolonged inflammatory phase, including the existence of persistent infection, the infiltration of inflammatory cells (neutrophils, monocytes/macrophages, mast cells and T cells), the excessive levels of proinflammatory cytokines, chemokines, proteases, reactive oxygen species and senescent cells, as well as the release of ECM degradation enzymes such as matrix metalloproteases and collagenases [25, 26]. The underlying pathophysiological mechanisms relate to increased oxidative stress, diminished cell recruitment and proliferation, deficiency of growth factors, impaired formation of collagen matrix, and, most importantly, impaired angiogenesis and/or neovascularization [23, 27–31].

In the quest for the ideal treatment, the use of MSCs has been advocated, considering their role in wound healing and their overall self-renewable, immunomodulatory, anti-inflammatory, anti-fibrotic, angiogenic and therapeutic capacities [32–34]. The main molecular mechanism in MSC-mediated diabetic wound healing is that MSCs secrete angiogenic growth factors, immunomodulatory factors, remodelling molecules, and extracellular vesicles (EVs) to enhance re-epithelialization, granulation tissue formation, and neovascularization in the diabetic wound bed [35]. Rat adipose derived MSCs (AD-MSCs) were found to secrete angiogenic factors (vascular endothelial growth factor (VEGF), hepatocyte growth factor (HGF), and basic fibroblast growth factor (bFGF)) in vitro and in vivo, resulting in an increased neovascularization and enhanced wound closure in diabetic rat model [36]. MSCs can migrate and home to the wound area and adhere to endothelial cells via interferon gamma, tumour necrosis factor-α (TNF-α), C–C chemokine receptor type 7, intercellular adhesion molecule-1(ICAM-1), vascular cell adhesion molecule 1, and Akt-dependent mechanisms [37–39]. At the wound area, MSCs stimulate neovascularization, through interaction with VEGF, endothelial nitric oxide synthase and hypoxia-inducible factor (HIF) pathways [38], and immunomodulation, via interaction with T and B cells, macrophages and natural killer cells [40]. Intradermally injecting MSCs around diabetic wounds accelerated the wound closure, re-epithelialization and granulation tissue formation via secretion of chemokines, inflammatory cytokines and immune factors including TNF-α, interleukin (IL)-1, IL-6, IL-8, MCP-1, PEG2 and IL-10 [41–44]. MSCs inhibit the expression of matrix metallopeptidase(MMP)-1 and upregulate MMP-9 to suppress the degradation of collagen matrix and facilitate fibroblast and keratinocyte proliferation and migration across the wound bed [45]. EVs secreted by MSCs, containing proteins, microRNAs, coding RNAs and non-coding RNAs, and mitochondria, demonstrated a positive effect on diabetic wound healing as well. MSC-derived EVs containing long noncoding RNA H19 stimulate diabetic wound healing process through suppressing the apoptosis and inflammation of fibroblasts via miR-152-3p-mediated PTEN axis [46]. EVs derived from MSCs containing miR-126 activate the PI3K/AKT signalling pathway via downregulating PTEN, resulting in enhanced wound healing and angiogenesis in diabetic rat wounds [47]. EVs derived from MSCs containing either let-7b [48] or miR-211-3p [49], target TRL4/NF-κB/STAT3/AKT pathway and AKT/eNOS pathway, respectively, modulating immune response, inflammation and angiogenesis in diabetic preclinical wound models.

**Fig. 1:**
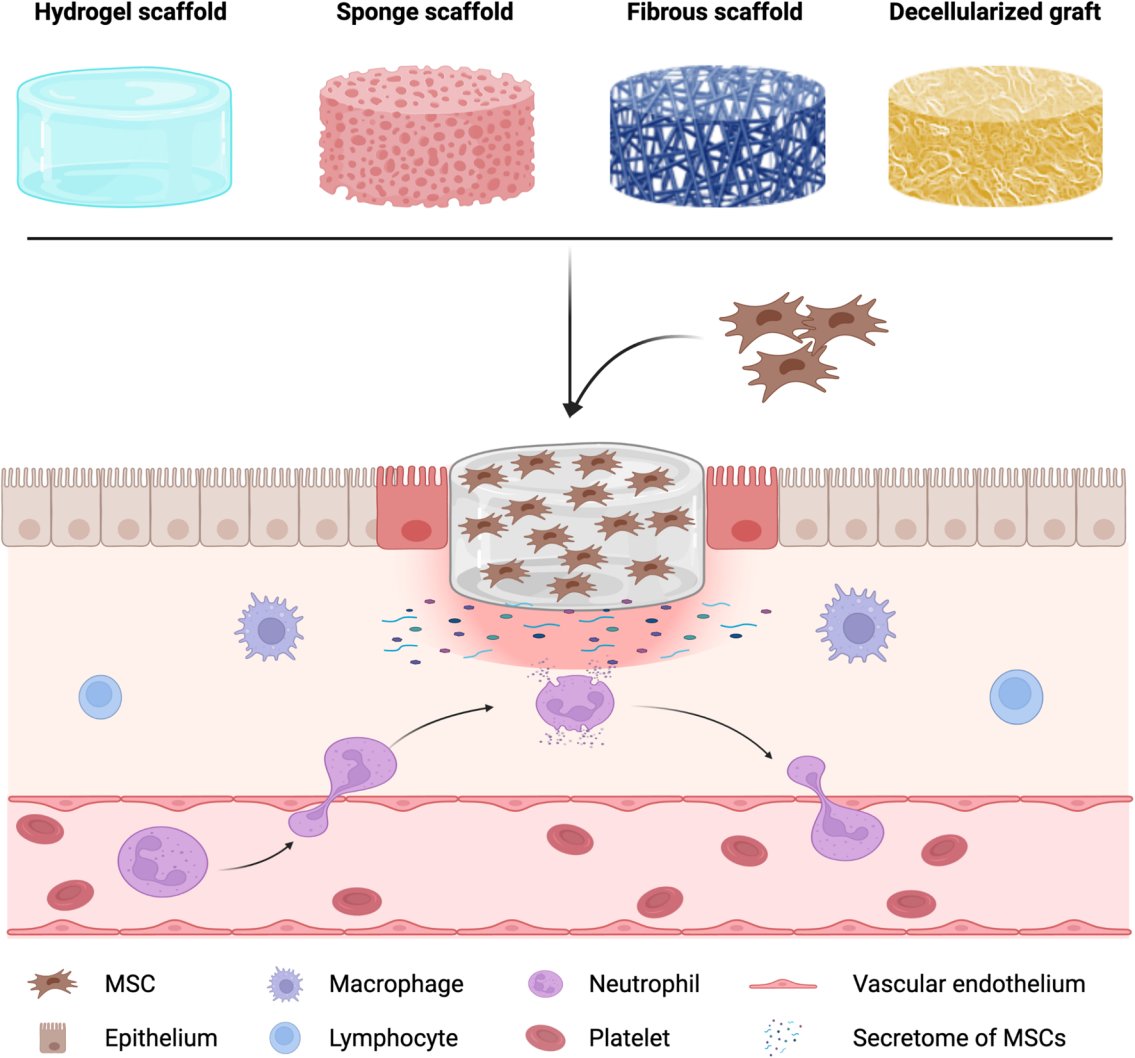
Summarized representations of various scaffolds used for MSC delivery. Enhanced delivery of MSC can be achieved using scaffolds and grafts that mimic or retain the architecture of natural human tissue, providing a favourable microenvironment for MSCs to attach, proliferate, and retain their secretome, as well as guide the host cell migration. The secretome of MSCs stimulate the infiltration and migration of immune cells (macrophages, lymphocytes, and neutrophil) that will modulate the inflammation and immune response in the wound bed, thus promoting angiogenesis and improving wound healing. From left to right, we depicted the main cell carriers used to delivery MSCs in diabetic wound healing studies. Hydrogel scaffolds hold a high fraction of water within its structure; sponge scaffolds exhibit highly uniform interconnected pore network; fibrous scaffolds consist of fibres at microscale or nanoscale level; and decellularized grafts retain their native ECM elements and anatomical structure. (Created in BioRender.com)

Although MSCs have been shown to improve wound healing, their short time in the wound bed prevents the full realization of their therapeutic potential and has triggered investigations of the optimal MSC carrier system (Fig. 1, Table 1).

**Table 1:** Fabrication method, benefits and limitations of different scaffolds for cell delivery

Scaffold formation	Fabrication method	Benefits	Limitations
Hydrogel scaffold	Physical/chemical cross-linking	Highly biocompatible and biodegradable	Natural hydrogels do not have strong mechanical strength, require combining with synthetic ones. Batch-to-batch variation
	Polymerization grafting	Low cytotoxicity	
	Radiation cross-linking	Similarity to physiological environment in human tissue	
Sponge scaffold	Freeze-drying	The uniform interconnected pore network provides suitable microenvironment for cell attachment, migration, and nutrient transition	The surface and pore structures require to be adjusted based on cell types and host tissue
	Gas foaming	The swelling capacity of scaffold influence cell behaviour and allow absorption of the exudate in the wound	The fabrication procedure is time consuming
	Porogen leaching		
Fibrous scaffold	Electrospinning	Mimic the micro- or nano- structure of human tissue	Small pore size of fibrous scaffolds may hamper cellular migration, restricting tissue ingrowth
	Fibre bonding	High surface-area-to-volume ratio is suitable for cell adhesion, proliferation, migration, and differentiation	
	Needle punch	Flexible mechanical properties	
Decellularized graft	Physical methods (freezing, force etc.)	Retained native ECM component and structure are favourable for cell attachment, migration, and differentiation	Complete decellularization is essential to avoid immune response
	Chemical methods (acid, Triton etc.) Enzymatic methods (Trypsin, pepsin etc.)	Higher mechanical strength	

## Hydrogel scaffolds for MSC delivery in diabetic models

Hydrogels are three-dimensional networks comprised of natural, synthetic or combinations of polymers that have the ability to swell and hold a significant fraction of water within their structure (Fig. 1). Hydrogels have received considerable attention in therapeutic approaches to wound healing, due to their ability to maintain cell viability at the implantation site and flexibility of fabrication [50, 51]. The naturally derived hydrogels have shown several advantages: biocompatibility, biodegradability, intrinsic cellular interactions, and structural similarity to the natural human tissue [52]. In contrast, the limitations of natural hydrogels include a narrow range of mechanical properties and batch variability [53]. Therefore, natural and synthetic hydrogels are often combined to create composite hydrogels with controlled structure and function [54]. Composite hydrogel scaffold designs have attracted significant attention since their properties of being able to be engineered with controllable shape, size, surface competence, biodegradation and biocompatibility, which suit the mechanical and biomedical requirements for wound healing and skin regeneration [55]. Several hydrogel systems have been assessed as MSC carriers in diabetic wound healing models (Table 2). In spite of this extensive literature, which is reviewed here, progression to the clinic is limited. As clinical translation is the goal of preclinical research, in this section, studies conducted using MSCs from different species were summarised separately to demonstrate the current research status based on the species of origin of the transplanted cells.

**Table 2:** Summary of scaffolds for MSC delivery in diabetic wound healing

Scaffold formation	Material	Cell type	Animal model	Outcome	Possible mechanism	References
Hydrogel scaffold	Collagen type I	Mouse BM-MSCs and AD-MSCs	STZ-induced diabetic C57BL/6 mouse	Murine BM-MSCs and AD-MSCs are equivalent at enhancing wound healing	MSC treatment improve wound healing by increasing VEGF-A expression, cellular proliferation, endothelial cell density, numbers of macrophages and smooth muscle cells and upregulating Notch signalling	Guo et al. [57]
Hydrogel scaffold	Collagen type I	Mouse BM-MSCs	STZ-induced diabetic C57BL/6 mouse	Transplantation of rolled scaffolds containing BM-MSC increased wound healing, cellular proliferation and capillary density as well as increased number of macrophages, fibroblasts and smooth muscle cells	Scaffolds in a rolled formation, were hypoxia, induced MSC secrete VEGF	Assi et al. [56]
Hydrogel scaffold	PEGDA and gelatin	Mouse AD-MSCs	*db/db* diabetic mouse	AD-MSC embedded hydrogel significantly accelerated diabetic wound healing	Hydrogel-mediated delivery of AD-MSCs accelerated wound closure by supressing infiltration of inflammatory cells (macrophages and T cells) and enhancing neovascularization	Dong et al. [59]
Hydrogel scaffold	N-isopropylacrylamide and poly (amidoamine)	Mouse BM-MSCs	*db/db* diabetic mouse	Promoted granulation tissue formation, angiogenesis, ECM secretion, wound contraction, and re-epithelialization	Hydrogel promoted BM-MSC secretion of TGFβ-1 and bFGF, inhibited pro-inflammatory M1 macrophage expression	Chen et al. [60]
Hydrogel scaffold	Pluronic F-127	Rat AD-MSCs	STZ-induced diabetic Sprague Dawley rat	AD-MSC-hydrogel enhanced angiogenesis and cell proliferation at the wound site, accelerated wound closure	Upregulated expression of VEGF and TGFβ-1 play a key role in matrix deposition, cellular migration and wound healing	Kaisang et al. [62]
Hydrogel scaffold	Silk fibroin, chitosan	Rat AD-MSCs	STZ-induced diabetic Sprague Dawley rat	Wound closure rate increased. Neovascularization improved	AD-MSCs engrafted in hydrogel scaffold promoted the secretion level of EGF, TGF-β, VEGF in the diabetic wound bed	Wu et al. [63]
Hydrogel scaffold	POLY(*β*-aminoester)-tetraaniline, HA, gelatin, laccase	AD-MSCs (unknown species)	STZ-induced diabetic Sprague Dawley rat	AD-MSC encapsulated hydrogel enhanced vascular regeneration and immunoregulation in diabetic wound bed, promoted the reconstruction of blood vessels, hair follicles and dermal collagen matrix	AD-MSCs encapsulated in hydrogel exhibited upregulated expression of HIF-1*a* and connexin 43	Jin et al. [66]
Hydrogel scaffold	HA and PEGDA	AD-MSCs (species unknown)	STZ-induced diabetic Sprague Dawley rat	Improved diabetic wound healing process, enhanced angiogenesis and re-epithelialization	Hydrogel maintained the stemness and secretion abilities of AD-MSCs	Xu et al. [67]
Hydrogel scaffold	Chitosan and HA	Rat BM-MSCs	STZ-induced diabetic Sprague Dawley rat	Promoted granulation tissue formation, collagen deposition, cell proliferation, neovascularization and enhanced diabetic wound healing	The secretion of growth factors (TGF-β1, VEGF and bFGF) from BM-MSCs were increased, hydrogel regulated the inflammatory environment via modulating the macrophages polarization	Bai et al. [64]
Hydrogel scaffold	Chitosan, polyvinyl alcohol, S-nitroso-N-acetylpenicillamine (SNAP)	Rabbit BM-MSCs	Alloxan-induced diabetic rabbit	SNAP-loaded hydrogel combined with BM-MSC significantly improved wound healing rate, re-epithelialization and collagen deposition	The gene-expression of VEGF and SDF-1a were significantly upregulated in wounds treated with BM-MSCs embedded in Nitric-oxide-releasing hydrogels	Ahmed et al. [65]
Hydrogel scaffold	HA	Human AD-MSCs	*db/db* diabetic mouse	AD-MSC promoted wound closure and accelerated epithelialization	Stem cell markers (NANOG, OCT3/4, SOX-2 and SSEA-3) were up-regulated in AD-MSC microgel	Feng et al. [68]
Hydrogel scaffold	Gellan gum and HA	Human AD-MSCs	STZ-induced diabetic CD1-ICR mouse	AD-MSCs treatment resulted in re-epithelialization, thicker and more differentiated epidermis on wound bed	AD-MSCs treatment improve wound healing via modulating the inflammatory response during proliferative phase of wound healing to promote a successful neovascularization	Da silva et al. [70]
Hydrogel scaffold	Decellularized adipose matrix	Human AD-MSCs	KK/Upj-Ay/J mouse (Diabetic mouse)	Accelerated wound closure and increased neovascularization	Decellularized adipose matrix supported hAD-MSCs survival and proliferation, enhanced paracrine activity and increased secretion of HGF	Chen et al. [71]
Hydrogel scaffold	HyStem®-HP hydrogel	VEGFA hyper secreted human BM-MSCs	*db/db* diabetic mouse	Improved wound healing rate in wounds treated with VEGFA hyper secreted hBM-MSCs	N/A	Srifa et al. [72]
Hydrogel scaffold	PEGDA, 1-vinyl-2-pyrrolidinone, eosin Y	Rat ISCs and human BM-MSCs	*db/db* diabetic mouse	ISC:MSC combination group accelerate diabetic wound healing almost 3 times faster than control group (14 vs. ~ 40 days), without intermediate scab or scar	Co-encapsulation of ISC and BM-MSC in hydrogel improved wound healing by secreting insulin, VEGF, and TGFβ-1. The viability and function of MSC improved due to activation of the PI3K-Akt/PKB pathway	Aijaz et al. [73]
Sponge scaffold	Collagen type I	Rabbit BM-MSCs	Alloxan-induced diabetic rabbit	Collagen BM-MSC treatment promoted wound closure and angiogenesis in diabetic rabbit ulcer	Increased total length of blood vessels with enhanced neovascularization in collagen BM-MSC group	O’Loughlin et al. [79]
Sponge scaffold	Collagen, chitosan	Rat BM-MSCs	STZ-induced diabetic Wistar rat	BM-MSC treatment accelerated wound closure in diabetic rat	Hypoxia pre-treated BM-MSC increased the expression of HIF-1a, VEGF, and PDGF, promoted wound closure via reducing inflammation and enhancing angiogenesis in diabetic wound bed	Tong et al. [80]
Sponge scaffold	Chitosan, collagen, nanostructured lipid carriers, simvastatin	Rat epidermal MSCs	STZ-induced diabetic Wistar rat	Increased wound closure rate, promoted vascularization, enhanced viability and proliferation of stem cells	Sponge scaffolds provide a microenvironment suitable for cell proliferation, molecules transmission, and a controlled release of simvastatin	Örgül et al. [82]
Sponge scaffold	Chitosan and polyurethane	Rat AD-MSCs	STZ-induced diabetic Sprague Dawley rat	MSC-scaffold bio-complex and acupuncture treatment improved wound closure (90.34 ± 2.3%), completely re-epithelialized in 8 days	The combined treatment of MSC-scaffold bio-complex and acupuncture on wounds produced synergistic immunomodulatory effects via activating C3a and C5a, up-regulating the secretion of cytokines SDF-1 and TGF β-1, and downregulating proinflammatory cytokines TNF-α and IL-1β	Chen et al. [83]
Sponge scaffold	Curcumin, chitosan, alginate, EGF	Mouse BM-MSCs	STZ-induced diabetic Sprague Dawley rat	BM-MSCs delivered by Curcumin-EGF scaffold significantly improved wound closure by increasing granulation tissue formation, collagen deposition and angiogenesis	The scaffold enhanced BM-MSC viability and expression of transcription factors associated with the maintenance of pluripotency and self-renewal (OCT3⁄4, SOX2, and Nanog)	Mohanty et al. [84]
Fibrous scaffold	Polycaprolactone, pluronic-F-127, gelatin	Mouse BM-MSCs	TALLYHO type 2 diabetic mouse	BM-MSC engraftment enhanced granulation tissue formation, promoted angiogenesis and collagen deposition in diabetic wound site	The BM-MSC engraftment inhibited the formation of M1-type macrophages and expression of pro-inflammatory cytokines (IL-6, TNF-α), promoted formation of M2-type macrophages and expression of anti-inflammatory cytokines (IL-4, IL-10) in the diabetic wound	Chen et al. [92]
Fibrous scaffold	Polylactic acid, silk and collagen	Human BM-MSCs	STZ-induced C57BL/6 J diabetic mouse	HO-1-overexpressing human BM-MSCs-scaffold complex significantly promote angiogenesis and wound healing	Over-expression of HO-1 promoted the proliferation and paracrine (e.g. VEGF) activity of BM-MSC via Akt signalling pathway	Hou et al. [93]
Fibrous scaffold	Polylactic acid, silk and collagen	Human BM-MSCs	STZ-induced C57BL/6 J diabetic mouse	Wound healed prominently, more blood vessel formation	Brain-derived neurotrophic factor activated MSCs differentiate into endothelial cells and accelerated wound healing	He et al. [94]
Fibrous scaffold	Aloe vera, polycaprolactone	Human UC-MSCs	*db/db* diabetic mouse	Diabetic wounds showed rapid wound closure, re-epithelialization and increased number of sebaceous glands and hair follicles	After treatment, the wounds showed positive keratinocyte markers (cytokeratin, involucrin, filaggrin) and increased expression of ICAM-1, TIMP-1, and VEGF-A	Tam et al. [95]
Fibrous scaffold	Silk fibroin	Human AD-MSCs	*db/db* diabetic mouse	Both AD-MSCs-SF and decellularized AD-MSCs-SF significantly enhanced wound closure, completing the process in around 10 days as compared to 15–17 days in control group	SF bind angiogenic factors (bFGF and TGF-β) produced by AD-MSCs; AD-MSCs-SF stimulate hUVECs migration through release of VEGF; Enhanced ECM deposition, angiogenesis and immunomodulation; Down-regulated inflammatory gene expression (Mif and Il6st)	Navone et al. [96]
Decellularized graft	Cadaveric skins of human donors	Rat AD-MSCs	STZ-induced diabetic Sprague Dawley rat	AD-MSCs-scaffold treatment significantly enhanced wound closure and epithelialization	AD-MSCS secreted angiogenic growth factors (VEGF, HGF, TGFβ and bFGF) resulting in accelerated wound healing	Nie et al. [111]
Decellularized graft	IRC mouse skin	Mouse BM-MSCs	Diabetic ICR mouse	BM-MSC-decellularized graft increased angiogenesis and reepithelialisation on diabetic wound bed	BM-MSCs-scaffold treatment enhanced synthesis of collagen type I during wound healing, increased epidermal thickness and vessel density	Chu et al. [98]
Decellularized graft	IRC mouse skin	Mouse BM-MSCs	STZ-induced IRC mouse	Induced robust vascularization and collagen deposition and rapid re-epithelialization	Scaffolds provide a microenvironment for cell attachment, migration and proliferation	Fu et al. [112]
Decellularized graft	Porcine skin, collagen, and chitosan	Human UC-MSCs	STZ-induced diabetic Sprague Dawley rat	UC-MSC delivered by decellularized graft significantly improved wound healing	Therapeutic effect of UC-MSCs on diabetic wound significantly enhanced by the activation of Wnt signalling pathway	Han et al. [113]

### Hydrogel scaffolds for mouse-MSC delivery

A few studies investigated the therapeutic effect of mouse-MSCs delivered via different composited hydrogels to diabetic mouse wound models. It should be noted that delivery of mouse cells to mice will avoid any potential xenogeneic response when human derived cells are used. However, from a translational perspective this may create problems as the final product will use human cells which will not then be studied. There will be a need to show that the animal cells are identical to the human cells. Collagen is the most abundant protein in skin and has been considered as the first choice cell delivery platform for diabetic wound healing. Mouse bone marrow-derived MSCs (BM-MSCs) and AD-MSCs delivered with a type I rat tail collagen hydrogel enhanced wound healing in a diabetic mouse model by increasing growth factor expression (e.g. VEGF), and recruiting macrophages to modulate immune and inflammatory responses in the wound bed [56, 57]. The wound closure rate was significantly increased compared with the collagen alone group, suggesting that collagen hydrogel successfully delivered MSCs to the wound bed and enhanced the therapeutic outcome. Gelatin is a matrix metalloproteinase sensitive biodegradable biomacromolecule that is derived from collagen. When gelatin was combined with poly(ethylene glycol) (PEG), the hydrogel demonstrated a tuneable degradation speed depending on cell number and material concentration; when the right conditions were defined, the degradation process took place in parallel with the wound healing process [58]. Mouse AD-MSCs delivered by a gelatin hydrogel crosslinked by hyperbranched poly(ethylene glycol) diacrylate (PEGDA), demonstrated excellent cell attachment and maintained cell proliferation, cell viability and metabolic activity for 3 weeks. After injecting the AD-MSC-hydrogel on the wound surface of *db/db* diabetic mouse, cell retention in the wound bed was significantly improved, resulting in enhanced wound closure and neovascularization and reduced inflammation compared with no-treatment, cell alone and hydrogel alone controls [59]. In addition, mouse BM-MSCs were delivered via a biodegradable n-isopropylacrylamide-based, thermosensitive hydrogel to treat wounds in a *db/db* mouse model, resulting in enhanced extracellular matrix (ECM) deposition, neovascularization, re-epithelialization and granulation tissue formation via modulation of the polarization of M1 and M2 macrophages in the wound bed compared with no-treatment and hydrogel alone controls [60]. This thermosensitive hydrogel has been shown to promote the secretion of transforming growth factor-β (TGFβ)-1 and bFGF in BM-MSCs in vitro, which maybe the underlying mechanism of MSCs promoting diabetic wound healing.

### Hydrogel scaffolds for rat-MSC delivery

Pluronic F-127, a synthetic and biocompatible hydrogel, has been extensively investigated in the applications of drug delivery and controlled release in the past decade [61]. The unique characteristic of thermosensitivity, enables Pluronic F-127 hydrogel to easily encapsulate large numbers of cells and be delivered to the wound bed. Rat AD-MSCs encapsulated in Pluronic F-127 hydrogel significantly accelerated wound closure by enhanced angiogenesis and cell proliferation at the wound site in a streptozotocin (STZ) induced diabetic rat model [62]. Compared to no treatment, cell alone and hydrogel alone control groups, relative mRNA expression levels of key angiogenic (VEGF), and wound healing growth factors (TGF-β1) were upregulated in the MSC-hydrogel treated wounds, suggesting that MSC-hydrogel engraftment promoted wound healing via paracrine mechanisms. Rat AD-MSCs engrafted in silk-fibroin/chitosan hydrogel significantly improved re-epithelialization and granulation tissue formulation and capillary formation in diabetic rat wound bed after 7 days of treatment compared with the no-treatment and scaffold alone groups [63]. After 14 days of hydrogel-MSC treatment, the protein expression level of epidermal growth factor (EGF), TGF-β and VEGF in the wound bed were significantly increased compared with the non-treatment and scaffold alone groups. In addition, rat BM-MSCs delivered by a hydrogel consist of *N*-chitosan and HA to the wound bed of diabetic rats, inhibited chronic inflammation, promoted granulation tissue formation, collagen deposition and nucleated cell proliferation, and stimulated neovascularization, which resulted in enhanced diabetic wound healing. In vitro assessment revealed this HA-based hydrogel promoted the secretion of TGF-β1, VEGF and bFGF of BM-MSC [64].

### Hydrogel scaffold for rabbit-MSC delivery and others

Rabbit BM-MSCs delivered by a nitric-oxide-releasing hydrogel significantly improved wound healing rate, re-epithelialization, collagen deposition and upregulated the gene-expression of VEGF and stromal cell-derived factor(SDF)-1α in the wound bed of a diabetic rabbit model compared with no-treatment, cell alone and hydrogel alone controls [65].

In another study, AD-MSCs (unknown species) delivered by hyaluronic acid (HA) based composite hydrogel to diabetic rat wound, promoted the reconstruction of blood vessels, hair follicles and dermal collagen matrix, via the maintenance of stemness of MSC and upregulation of the gene expression of HIF-1*α* and connexin 43 in the wound bed compared with no-treatment, MSC alone and hydrogel alone controls [66, 67]. However, it is important to acknowledge the importance of specifying the species of MSC used in pre-clinical research in order to understand the full implications of the results. Moreover, this is key to consider the potential translation in clinical settings from early stages of experimental design and product development.

### Hydrogel scaffold for human-MSC delivery

In terms of hydrogel mediated human MSC delivery, subcutaneous injection of human AD-MSC micro-hydrogel prepared by HA improved wound healing in *db/db* mouse and resulted in faster wound epithelialization with thicker dermis formation compared with no-treatment, and hydrogel alone groups [68]. In this AD-MSC-hydrogel construct, the expression of stemness markers (NANOG, OCT3/4, SOX-2 and SSEA-3) at the protein level were significantly up-regulated compared with cells in monolayer culture, suggesting that this hydrogel mimicked a physiological microenvironment that promoted cell growth and induced a stemness-like phenotype [68, 69]. Human AD-MSCs have also been delivered through a combination of HA and gellan gum hydrogel for the treatment of diabetic mouse wounds. In this study, an improved impact on the neovascularization of diabetic wounds was observed and the epidermis of the healed diabetic wound was shown to be thicker and more differentiated than no-treatment control [70]. Decellularized adipose matrix, after freeze-drying, digestion with pepsin and neutralization, can also serve as a hydrogel for human AD-MSC delivery [71]. This thermosensitive hydrogel presented similar structural and biochemical complexity of native ECM, supported human AD-MSCs survival and proliferation, increased the MSC paracrine secretion (HGF), eventually enhanced the wound closure and neovascularization compared to local injection of AD-MSCs in a diabetic mouse model. On the other hand, researchers have applied engineered VEGFA-hypersecreting human BM-MSCs to *db/db* mouse wound bed by either direct injection or embedding cells in HyStem®-HP hydrogel. The results showed that both cell delivery methods improved wound healing rate, with a significant difference observed from 7–9 days after treatment, and the cells delivered by the hydrogel group showed similar healing kinetics compared to the direct injection group [72]. Another study reported that encapsulating a mixture of human BM-MSCs and rat insulin secreting cells (ISCs) in a PEGDA hydrogel promoted diabetic wound healing almost 3 times faster than control group (14 vs. ~ 40 days), without intermediate scab or scar, through increased secretion of insulin, VEGF, TGF-β1 and the viability and function of MSC improved due to activation of the PI3K-Akt/PKB pathway [73]. This observation suggests that a combination of different cell types may further enhance the therapeutic effects in the diabetic wound.

## Sponge scaffolds for MSC delivery in diabetic models

Sponge scaffolds are fabricated by natural or synthetic polymers via various techniques (e.g. porogen leaching, gas foaming and freeze-drying methods) and exhibit high porosity and a uniform interconnected pore network (Fig. 1) [74, 75]. Sponge scaffolds for tissue engineering can be described using several criteria, including pore size, porosity, water uptake and retention capacity [76]. The major difference between sponge and hydrogel scaffolds is the fabrication method which results in a difference in the percentage of water content in the scaffold. Compared to hydrogel, the fabrication procedure of sponge scaffolds is time consuming, and the surface and structure require to be adjusted depending on cell type and host tissue. Sponge scaffolds hold several potential advantages for skin wound healing. The highly porous structure of sponge scaffolds mimics the architecture of ECM supporting cells to migrate to the site of the defect [77]. The water uptake and retention capacity of sponge scaffolds allows them to absorb the exudate in the wound bed and provides a favourable environment for cell migration and proliferation [78].

In terms of utilisation of sponge scaffolds in diabetic wound healing applications, collagen and chitosan-based sponge scaffolds are the most commonly used MSC carriers. O’Loughlin, et al. have developed a collagen sponge scaffold using a freeze-drying method. Compared to no-treatment control, the topical administration of allogeneic BM-MSCs through a collagen sponge scaffold augmented wound closure and increased angiogenesis after transplantation for 7 days in the diabetic rabbit wound [79]. A collagen-chitosan sponge scaffold was constructed using cross-linking and a freeze-drying method, resulting in a 100 μm network pore configuration and a suitable swelling ratio and appropriate biodegradability for BM-MSC delivery [80]. This sponge scaffold provided a microenvironment whereby hypoxia pre-treated rat BM-MSCs secreted higher levels of proangiogenic factors such as VEGF and platelet-derived growth factor (PDGF) and upregulated the expression of key transcription factors such as HIF-1α, while maintaining cell viability. Transplantation of this BM-MSC-scaffold construct to a STZ-induced diabetic rat wound model, resulted in improved wound closure, increased angiogenesis and decreased inflammation (enhanced gene and protein expression of anti-inflammatory cytokine IL-10 at 7 and 14 days) in the wound bed compared to scaffold alone group. In addition, researchers developed a chitosan-collagen scaffold containing simvastatin that exhibited high porosity (pore size in a range of 20–200 μm), suitable mechanical strength with similar elasticity as human skin (83.3 ± 34.9 MPa) [81] and a controlled release of simvastatin. Rat epidermal-derived MSCs delivered by this scaffold resulted in increased wound closure rate, promoted vascularization, enhanced viability and proliferation of MSCs in diabetic rat wound compared to no-treatment control and scaffold alone group [82]. In another study, sponge scaffold consisting of glycol chitosan and polyurethane delivering rat AD-MSCs to STZ-induced diabetic rat wound, and combined with acupuncture produced synergistic immunomodulatory effects, resulted in improved wound closure (90.34 ± 2.3%) and complete re-epithelialization in 8 days than AD-MSC alone group [83]. The secretion of cytokines SDF-1 and TGF β-1 were upregulated and proinflammatory cytokines TNF-α and IL-1β were downregulated in the wound bed after 8 days treatment. Furthermore, sponge scaffolds can also conjugate with growth factors as a cell delivery system. Chitosan-alginate sponge scaffolds conjugated with EGF delivering BALB/c mouse BM-MSCs, resulted in enhanced cell viability and expression of transcription factors associated with the maintenance of pluripotency and self-renewal (OCT3⁄4, SOX2, and Nanog) in vitro, and showed significant improvement of wound closure, by increasing granulation tissue formation, collagen deposition and angiogenesis in diabetic rat wound as compared to no-treatment control and MSC alone groups [84].

## Fibrous scaffolds for MSC delivery in diabetic models

Fibrous scaffolds are mainly developed by an electrospinning method to create three-dimensional constructs consisting of fibres at microscale or nanoscale level to mimic the architecture of natural human tissue (**Fig. ****1**) [85–87]. Electrospinning is a technique that has been investigated for decades, by using electrostatic forces to produce continuous fibres from biocompatible materials [88]. The alignment of a fibrous scaffold can be random or aligned depending on the requirements for application. Other techniques, such as fibre bonding and needle punch, have also been used for fabrication of fibrous scaffolds [89]. Fibrous scaffolds have been employed in various tissue engineering fields, including bone, cartilage, skin, vascular and neural tissue engineering [90]. The high surface-area-to volume ratio of fibrous scaffolds allows cell adhesion, however small pore size may hinder cell migration and need to be adjusted based on cell type [91]. In recent years, there has been an increasing number of publications using fibrous scaffolds in wound healing applications due to their ability to serve as a structural template, improve cell–cell and cell–matrix interactions, and to direct cell behaviour and function (e.g. cell morphology, cell proliferation, differentiation) [77, 90].

In terms of cell delivery, fibrous scaffolds have been used as MSC carriers for diabetic wound healing. Chen et al. have developed a three-dimensional scaffold using polycaprolactone, pluronic-F-127 and gelatin to deliver mouse BM-MSC. Compared with no-treatment and scaffold alone controls, this fibrous scaffold-MSC construct resulted in enhanced granulation tissue formation, angiogenesis and collagen deposition in the wound bed of diabetic mouse, through modulating the polarization of macrophages and expression of inflammatory cytokines [92]. This radially and vertically aligned fibrous scaffold has size and shape characteristics which can be tailored to be suitable for various wounds. In addition, a hybrid electrospinning nanofiber scaffold containing 80% polylactic acid, 10% silk and 10% collagen was developed as a cell carrier to deliver HO-1-overexpressing human BM-MSCs to the diabetic mouse wound bed, resulting in significantly enhanced angiogenesis and wound healing via Akt signalling pathways [93]. Researchers have also used this platform to deliver brain-derived neurotrophic factor activated human BM-MSCs to the wound bed in a diabetic mouse model. Significantly accelerated wound closure and enhanced blood vessel formation on the wound bed was observed with the underlying mechanism potentially related to milieu-dependent differentiation [94]. Moreover, a fibrous scaffold made of aloe vera and polycaprolactone was developed to deliver human umbilical cord-derived MSCs (UC-MSCs) or their conditioned medium to the *db/db* mouse wound bed, with both treatments showing rapid wound closure, re-epithelialization and increased number of sebaceous glands and hair follicles after transplantation to the wounds for 28 days, no significant difference was observed between the two treatments throughout the study period [95]. After both treatments the wound showed positive keratinocyte markers and increased cytokine expression of ICAM-1, tissue inhibitor matrix metalloproteinase 1 (TIMP-1), and VEGF-A at day 14 and 28. Furthermore, a silk fibroin (SF) scaffold delivering human AD-MSCs to a *db/db* mouse wound model, resulted in complete wound closure at 10 days versus 15–17 days for controls [96]. The same therapeutic effects were observed even after removing MSCs from the SF-MSC bio-complex, indicating that the MSC secretions stored in the scaffold play a key role in improving the wound healing process.

## Decellularized grafts for MSC delivery in diabetic models

Decellularized grafts are mainly derived from tissues or organs through mechanical (freezing, force etc.), chemical (acid, Triton etc.) or enzymatic (trypsin, pepsin etc.) decellularization procedures to remove cellular components [97]. Commonly used tissue or organs include skin [98], Wharton's jelly [99] adipose tissue [100] and in vitro cultured cells [101]. Compared to other synthetic scaffolds, decellularized grafts are non-immunogenic and retain their native ECM elements (e.g. collagen, elastin, laminin and fibronectin), and anatomical structure (Fig. 1) [100–102]. These advantages are essential in identifying and developing scaffolds for implantation in diabetic wounds. Applications of decellularized grafts can replace the impaired ECM of diabetic wounds, providing ECM proteins such as collagen, glycosaminoglycans, proteoglycans and glycoproteins, allowing host cells to infiltrate, modulate the immune response and promote angiogenesis and the formation of granulation tissue [103, 104]. There are several commercially available decellularized grafts for wound healing, Integra (Johnson & Johnson) [105], Oasis (Cook Biotech) [106], Alloderm (Allergan) [107], Primatrix (TEI Bioscience) [108]. Their manufacturing methods vary resulting in different mechanical properties of each product and the ability to support skin regeneration [109, 110].

Several studies have investigated the use of decellularized grafts as a MSC delivery platform. One of these studies has shown that rat AD-MSCs seeded on a decellularized graft secreted various cytokines (e.g. HGF, VEGF, TGFβ, bFGF) that stimulated the migration and proliferation of fibroblasts, eventually resulting in improved wound closure (13 ± 0.37 days compared with 20 ± 0.71 days in scaffold alone group and 27 ± 0.44 days in no-treatment group) in a diabetic rat model [111]. In another study, mouse BM-MSCs were delivered by a graft that was decellularized from normal mouse skin. After transplanting this bio complex into a full-thickness cutaneous wound site in the diabetic mouse, the wound demonstrated an increased percentage of wound closure and significantly accelerated angiogenesis and reepithelialisation compared with no-treatment controls. The synthesis of collagen type I fibres was seen to be increased during diabetic wound healing, monitored using a novel multiphoton microscopy, indicating a potential mechanism of wound healing [98]. Decellularized dermal matrix incorporating reduced graphene oxide as a cell delivery platform has shown high stability and strong mechanical properties. This decellularized graft has been used to deliver mouse BM-MSCs to a diabetic mouse wound model resulting in a microenvironment for stem cell attachment, migration and proliferation, with robust vascularization and collagen deposition during wound healing [112]. Furthermore, human UC-MSCs delivered by a decellularized dermal matrix to the diabetic rat wound showed that the proliferation and differentiation of human UC-MSCs on the decellularized dermal matrix were regulated by activated Wnt signalling pathway, ultimately promoting the healing of the diabetic wound [113].

## Clinical trials using scaffold-mediated delivery of MSCs for diabetic wound healing

Currently, 13 clinical trials using scaffold-based delivery of MSCs to treat diabetic patients with foot ulcers are registered with ClinicalTrials.gov (Table 3). 11 of these trials are using a hydrogel scaffold for MSC delivery, and 2 are using a sponge scaffold. In spite of the very large pre-clinical dataset available and the 13 registered clinical trials of scaffold mediated MSC delivery, only one clinical study has been published to our knowledge. In this phase 2 clinical trial (No. NTC02619877), an allogeneic AD-MSC hydrogel sheet has been developed and approved to be a commercial product by Food and Drug Administration of Korea (study code ALLO-ASC-DFU-201). 59 patients with diabetic foot ulcers were enrolled in this trial for a maximum of 12 weeks. Patients treated with this MSC-hydrogel sheet reached 82% of complete wound closure at week 12 compared to 53% of complete wound closure in patients without treatment. No adverse effects were observed after treatment, indicating hydrogel delivered AD-MSCs is efficient, effective and safe in the treatment of diabetic wounds [114]. An important feature of this MSC-hydrogel graft was the ability to cryopreserve and store while maintaining, stability for long periods of time [114]. In another case study, placenta derived MSCs (cell number: 1 × 10^6^ cells/cm^2^) encapsulated in a sodium alginate hydrogel was topically administrated to the foot ulcer of a patient with type 2 diabetes mellitus. The wound healed completely after 3 weeks of treatment, with improved foot pain, no toxicity and no relapse during the subsequent 6 months follow-up visit [115]. However, this is only a case study and further evaluation is needed. In addition, there is one clinical study reported which evaluated the effect of a collagen sponge scaffold delivering MSC-like dermal autologous micro-grafts (obtained from mechanical disaggregation of small pieces of skin tissue) to diabetic patients with foot ulcers. These dermal micro-grafts express MSCs markers (CD34, CD73, CD90 and CD105) in vitro and maintained their viability and proliferative property in the collagen scaffold. After applying this dermal micrograft-collagen scaffold bio-complex to the site of ulcers, the skin samples express increased level of insulin-like growth factor and TNF-β and decreased level of EGF, PDGF and their receptors compared with healthy skin samples. Treatment of these ulcers with this bio-complex resulted in improved wound closure and better quality of life for the patients [116].

**Table 3:** Current clinical trials using scaffolds delivering MSCs for diabetic foot ulcer (from clinicaltrials.gov, 7th January 2022)

Identifier	Trial name	Study phase	Cell type	Cell delivery method	Recruitment status	Sponsor	Location
NCT02394886	Safety of ALLO-ASC-DFU in the patients with diabetic foot ulcers	Phase 1	Allogeneic AD-MSCs	Hydrogel scaffold	Completed	Anterogen Co., Ltd	Korea
NCT02619877	Clinical study to evaluate efficacy and safety of ALLO-ASC-DFU in patients with diabetic foot ulcers	Phase 2	Allogeneic AD-MSCs	Hydrogel scaffold	Completed	Anterogen Co., Ltd	Korea
NCT03183726	A follow-up study to evaluate the safety of ALLO-ASC-DFU in ALLO-ASC-DFU-101 clinical trial	Phase 1	Allogeneic AD-MSCs	Hydrogel scaffold	Completed	Anterogen Co., Ltd	Korea
NCT03183804	A follow-up study to evaluate the safety of ALLO-ASC-DFU in ALLO-ASC-DFU-201 clinical trial	Phase 2	Allogeneic AD-MSCs	Hydrogel scaffold	Unknown	Anterogen Co., Ltd	Korea
NCT03370874	Clinical study to evaluate efficacy and safety of ALLO-SC-DFU in patients with diabetic foot ulcers	Phase 3	Allogeneic AD-MSCs	Hydrogel scaffold	Active, not recruiting	Anterogen Co., Ltd	Korea
NCT03754465	Clinical study of ALLO-ASC-SHEET in subjects with diabetic foot ulcers	Phase 2	Allogeneic AD-MSCs	Hydrogel scaffold	Recruiting	Anterogen Co., Ltd	United states
NCT04497805	Clinical study of ALLO-ASC-SHEET in subjects with diabetic wagner grade II foot ulcers	Phase 2	Allogeneic AD-MSCs	Hydrogel scaffold	Recruiting	Anterogen Co., Ltd	United states
NCT04569409	Clinical study to evaluate efficacy and safety of ALLO-ASC-DFU in patients with diabetic wagner grade 2 foot ulcers	Phase 3	Allogeneic AD-MSCs	Hydrogel scaffold	Recruiting	Anterogen Co., Ltd	Korea
NCT04464213	Human placental Mesenchymal stem cell treatment on diabetic foot ulcer	Phase 1	Placental MSCs	Hydrogel scaffold	Recruiting	Beijing Tongren Hospital	China
NCT03865394	Treatment of chronic wounds in diabetic foot syndrome with autologous adipose derived mesenchymal stem cells (1ABC)	Phase 1 Phase 2	Autologous AD-MSCs	Hydrogel scaffold	Completed	Medical University of Warsaw	Poland
NCT03248466	PRG combined with autologous BMMSCs for treatment of diabetic foot ulcer	Early phase 1	Autologous BM-MSCs	Hydrogel scaffold	Unknown	Third Military Medical University	China
NCT03259217	Clinical application of mesenchymal stem cells seeded in chitosan scaffold for diabetic foot ulcers	Phase 1	AD-MSCs	Sponge scaffold	Unknown	Assiut University	Korea
NCT02672280	Safety and exploratory efficacy study of collagen membrane with mesenchymal stem cells in the treatment of skin defects	Phase 1 Phase 2	UC-MSCs	Sponge scaffold	Unknown	South China Research Centre for Stem Cell and Regenerative Medicine	China

Interestingly, 9 out of the 13 clinical trials used AD-MSCs as a cell source despite a large number of pre-clinical studies reporting therapeutic benefits with the use of the three major sources (bone marrow, adipose and umbilical cord) [65, 95, 111]. This could be related to the feasibility of clinical translation, where all of them show advantages and disadvantages. BM-MSCs showed great therapeutic potential in wound healing and are suitable for autologous transplantation [117]. However, their isolation requires an invasive procedure and low cell numbers, limiting their clinical translation [118]. In recent years, UC-MSCs have gained more attention in the field as an alternative source; they can be easily isolated using non-invasive procedures and yield a large number of cells from a young donor [119]. Moreover, they have shown interesting therapeutic abilities due to their low immunogenicity and immuno-regulatory properties [120]. Akin to BM-MSCs, AD-MSCs have been shown to be ideal for autologous application; adipose tissue can be obtained with less invasive procedures and yield a higher number of cells, easily meeting clinical needs [121]. However, both sources are isolated from adult tissues and donor health status or other intrinsic factors, such as the site of tissue collection in the case of AD-MSC, could result in underlying effects upon cellular characteristics that hinder their therapeutic potential [119]. Future studies should consider in-depth investigation of head-to-head biological and therapeutic differences among these sources and whether these differences have implications in the context of diabetic wound management.

Besides defining optimal cell source, there are many other bottlenecks hindering the translation of results obtained at the laboratory bench to the clinic and ultimately to the marketplace. These include the absence of a globally shared standard manufacturing process and the high level of regulatory diversifications across countries which create crucial challenges for international clinical trial collaborations and cross-country marketing procedures [122]. In addition, the mainstream widely accepted treatment of neuropathic diabetic foot ulcers is debridement and off weight bearing and clinical trials do not always include state of the art current clinical treatment in the control group. This also leads to poor uptake by medical practitioners of advanced therapies. Finally, re-imbursement issues and cost of goods impact on the utilization of these advanced therapy products in standard clinical practice. It would be useful if researchers interested in clinical application consider the translational requirements from the earliest stages of research so that experiments can be designed in a manner suitable for inclusion in a regulatory dossier when applying for approval to undertake a clinical trial. There is also a need for careful clinical trial design as outlined previously by experts in the field [123, 124]. Partnerships with industry will also be crucial to enable translation.

## Conclusions

MSCs are attractive therapeutic agents for use in diabetic wound healing, whilst biodegradable scaffolds provide a physiologically relevant three-dimensional environment for their optimal growth and localisation at wound bed. Pre-clinical studies have demonstrated that delivering MSCs via a variety of different scaffolds to diabetic wounds accelerates healing and enhances skin regeneration. In spite of this, there is limited clinical trial data available on the use of scaffold-based MSC delivery for treatment of diabetic wounds. Issues regarding safety, efficacy and cost of the MSC-scaffold graft in clinical applications should be considered from an early stage of research and properly addressed. Therefore, an interdisciplinary approach involving biomedical scientists, clinicians, biomaterial engineers, industry and regulators will be necessary to develop a scaffold-based cell therapy product suitable for clinical application.

## Data Availability

Not applicable.

## Abbreviations

AD-MSCs: Adipose-derived mesenchymal stromal cells
BM-MSCs: Bone marrow-derived mesenchymal stromal cells
bFGF: Basic fibroblast growth factor
ECM: Extracellular matrix
EGF: Epidermal growth factor
EVs: Extracellular vesicles
HA: Hyaluronic acid
HGF: Hepatocyte growth factor
HIF: Hypoxia-inducible factor
ICAM-1: Intercellular adhesion molecule-1
IL: Interleukin
ISCs: Insulin secreting cells
MMP: Metallopeptidase
MSCs: Mesenchymal stromal cells
PDGF: Platelet-derived growth factor
PEG: Poly(ethylene glycol)
PEGDA: Poly(ethylene glycol) diacrylate
SDF: Stromal cell-derived factor
SF: Silk fibroin
STZ: Streptozotocin
TGF-β: Transforming growth factor-β
TIMP-1: Tissue inhibitor matrix metalloproteinase 1
TNF-α: Tumour necrosis factor-α
UC-MSCs: Umbilical cord-derived mesenchymal stromal cells
VEGF: Vascular endothelial growth factor

## References

[1] Ogurtsova K, Guariguata L, Barengo NC, Ruiz PL, Sacre JW, Karuranga S, Sun H, Boyko EJ, Magliano DJ (2021). IDF diabetes Atlas: global estimates of undiagnosed diabetes in adults for 2021. Diabetes Res Clin Pract.

[2] Sun H, Saeedi P, Karuranga S, Pinkepank M, Ogurtsova K, Duncan BB, Stein C, Basit A, Chan JCN, Mbanya JC, Pavkov ME, Ramachandaran A, Wild SH, James S, Herman WH, Zhang P, Bommer C, Kuo S, Boyko EJ, Magliano DJ (2021). IDF diabetes Atlas: global, regional and country-level diabetes prevalence estimates for 2021 and projections for 2045. Diabetes Res Clin Pract.

[3] Zhang P, Lu J, Jing Y, Tang S, Zhu D, Bi Y (2017). Global epidemiology of diabetic foot ulceration: a systematic review and meta-analysis (dagger). Ann Med.

[4] Singh N, Armstrong DG, Lipsky BA (2005). Preventing foot ulcers in patients with diabetes. JAMA.

[5] Consensus Development Conference on Diabetic Foot Wound Care. 7–8 April 1999, Boston, Massachusetts. American Diabetes Association, J Am Podiatr Med Assoc 89(9) (1999) 475–83. 10.7547/87507315-89-9-475 10507217

[6] Guest JF, Ayoub N, McIlwraith T, Uchegbu I, Gerrish A, Weidlich D, Vowden K, Vowden P (2017). Health economic burden that different wound types impose on the UK's National Health Service. Int Wound J.

[7] Sen CK, Gordillo GM, Roy S, Kirsner R, Lambert L, Hunt TK, Gottrup F, Gurtner GC, Longaker MT (2009). Human skin wounds: a major and snowballing threat to public health and the economy. Wound Repair Regen.

[8] Sen CK (2019). Human wounds and its burden: an updated compendium of estimates. Adv Wound Care (New Rochelle).

[9] Hocking AM (2012). Mesenchymal stem cell therapy for cutaneous wounds. Adv Wound Care (New Rochelle).

[10] Tchanque-Fossuo CN, Dahle SE, Lev-Tov H, West KIM, Li CS, Rocke DM, Isseroff RR (2019). Cellular versus acellular matrix devices in the treatment of diabetic foot ulcers: interim results of a comparative efficacy randomized controlled trial. J Tissue Eng Regen Med.

[11] Behm B, Babilas P, Landthaler M, Schreml S (2012). Cytokines, chemokines and growth factors in wound healing. J Eur Acad Dermatol Venereol.

[12] Chen L, Tredget EE, Wu PY, Wu Y (2008). Paracrine factors of mesenchymal stem cells recruit macrophages and endothelial lineage cells and enhance wound healing. PLoS ONE.

[13] Alfaro MP, Deskins DL, Wallus M, Das Gupta J, Davidson JM, Nanney LB, Gannon AGMM, Young PP (2013). A physiological role for connective tissue growth factor in early wound healing. Lab Invest.

[14] Maxson S, Lopez EA, Yoo D, Danilkovitch-Miagkova A, Leroux MA (2012). Concise review: role of mesenchymal stem cells in wound repair. Stem Cells Transl Med.

[15] Rustad KC, Gurtner GC (2012). Mesenchymal stem cells home to sites of injury and inflammation. Adv Wound Care (New Rochelle).

[16] El-Sadik AO, El-Ghamrawy TA, Abd El-Galil TI (2015). The effect of mesenchymal stem cells and chitosan gel on full thickness skin wound healing in albino rats: histological, immunohistochemical and fluorescent study. PLoS One.

[17] Marquardt LM, Heilshorn SC (2016). Design of injectable materials to improve stem cell transplantation. Curr Stem Cell Rep.

[18] Croll TI, Gentz S, Mueller K, Davidson M, O'Connor AJ, Stevens GW, Cooper-White JJ (2005). Modelling oxygen diffusion and cell growth in a porous, vascularising scaffold for soft tissue engineering applications. Chem Eng Sci.

[19] Edwards JV, Howley P, Cohen IK (2004). In vitro inhibition of human neutrophil elastase by oleic acid albumin formulations from derivatized cotton wound dressings. Int J Pharm.

[20] Frykberg RG, Banks J (2015). Challenges in the treatment of chronic wounds. Adv Wound Care (New Rochelle).

[21] Shah SA, Sohail M, Khan S, Minhas MU, de Matas M, Sikstone V, Hussain Z, Abbasi M, Kousar M (2019). Biopolymer-based biomaterials for accelerated diabetic wound healing: a critical review. Int J Biol Macromol.

[22] Pradhan L, Nabzdyk C, Andersen ND, LoGerfo FW, Veves A (2009). Inflammation and neuropeptides: the connection in diabetic wound healing. Expert Rev Mol Med.

[23] den Dekker A, Davis FM, Kunkel SL, Gallagher KA (2019). Targeting epigenetic mechanisms in diabetic wound healing. Transl Res.

[24] Ramirez-Acuna JM, Cardenas-Cadena SA, Marquez-Salas PA, Garza-Veloz I, Perez-Favila A, Cid-Baez MA, Flores-Morales V, Martinez-Fierro ML (2019). Diabetic foot ulcers: current advances in antimicrobial therapies and emerging treatments. Antibiotics (Basel).

[25] Holl J, Kowalewski C, Zimek Z, Fiedor P, Kaminski A, Oldak T, Moniuszko M, Eljaszewicz A (2021). Chronic diabetic wounds and their treatment with skin substitutes. Cells.

[26] Geng K, Ma X, Jiang Z, Huang W, Gao C, Pu Y, Luo L, Xu Y, Xu Y (2021). Innate immunity in diabetic wound healing: focus on the mastermind hidden in chronic inflammatory. Front Pharmacol.

[27] Davey GC, Patil SB, O'Loughlin A, O'Brien T (2014). Mesenchymal stem cell-based treatment for microvascular and secondary complications of diabetes mellitus. Front Endocrinol (Lausanne).

[28] Sharp A, Clark J (2011). Diabetes and its effects on wound healing. Nurs Stand.

[29] Mallik SB, Jayashree BS, Shenoy RR (2018). Epigenetic modulation of macrophage polarization- perspectives in diabetic wounds. J Diabetes Complicat.

[30] Zhao R, Liang H, Clarke E, Jackson C, Xue M (2016). Inflammation in chronic wounds. Int J Mol Sci.

[31] Veith AP, Henderson K, Spencer A, Sligar AD, Baker AB (2018). Therapeutic strategies for enhancing angiogenesis in wound healing. Adv Drug Deliv Rev.

[32] Hamdan S, Pastar I, Drakulich S, Dikici E, Tomic-Canic M, Deo S, Daunert S (2017). Nanotechnology-driven therapeutic interventions in wound healing: potential uses and applications. ACS Cent Sci.

[33] Li T, Ma H, Ma H, Ma Z, Qiang L, Yang Z, Yang X, Zhou X, Dai K, Wang J (2019). Mussel-inspired nanostructures potentiate the immunomodulatory properties and angiogenesis of mesenchymal stem cells. ACS Appl Mater Interfaces.

[34] Gu C, Huang S, Gao D, Wu Y, Li J, Ma K, Wu X, Fu X (2014). Angiogenic effect of mesenchymal stem cells as a therapeutic target for enhancing diabetic wound healing. Int J Low Extrem Wounds.

[35] An T, Chen Y, Tu Y, Lin P (2021). Mesenchymal stromal cell-derived extracellular vesicles in the treatment of diabetic foot ulcers: application and challenges. Stem Cell Rev Rep.

[36] Nie C, Yang D, Xu J, Si Z, Jin X, Zhang J (2011). Locally administered adipose-derived stem cells accelerate wound healing through differentiation and vasculogenesis. Cell Transpl.

[37] Sasaki M, Abe R, Fujita Y, Ando S, Inokuma D, Shimizu H (2008). Mesenchymal stem cells are recruited into wounded skin and contribute to wound repair by transdifferentiation into multiple skin cell type. J Immunol.

[38] Amin AH, Elmageed ZYA, Nair D, Partyka MI, Kadowitz PJ, Belmadani S, Matrougui K (2010). Modified multipotent stromal cells with epidermal growth factor restore vasculogenesis and blood flow in ischemic hind-limb of type II diabetic mice. Lab Invest.

[39] Hemeda H, Jakob M, Ludwig AK, Giebel B, Lang S, Brandau S (2010). Interferon-gamma and tumor necrosis factor-alpha differentially affect cytokine expression and migration properties of mesenchymal stem cells. Stem Cells Dev.

[40] Tatsumi K, Otani H, Sato D, Enoki C, Iwasaka T, Imamura H, Taniuchi S, Kaneko K, Adachi Y, Ikehara S (2008). Granulocyte-colony stimulating factor increases donor mesenchymal stem cells in bone marrow and their mobilization into peripheral circulation but does not repair dystrophic heart after bone marrow transplantation. Circ J.

[41] Liu W, Yu M, Xie D, Wang L, Ye C, Zhu Q, Liu F, Yang L (2020). Melatonin-stimulated MSC-derived exosomes improve diabetic wound healing through regulating macrophage M1 and M2 polarization by targeting the PTEN/AKT pathway. Stem Cell Res Ther.

[42] Singer NG, Caplan AI (2011). Mesenchymal stem cells: mechanisms of inflammation. Annu Rev Pathol.

[43] Sun Y, Song L, Zhang Y, Wang H, Dong X (2020). Adipose stem cells from type 2 diabetic mice exhibit therapeutic potential in wound healing. Stem Cell Res Ther.

[44] Bartaula-Brevik S, Pedersen TO, Blois AL, Papadakou P, Finne-Wistrand A, Xue Y, Bolstad AI, Mustafa K (2014). Leukocyte transmigration into tissue-engineered constructs is influenced by endothelial cells through Toll-like receptor signaling. Stem Cell Res Ther.

[45] Li H, Rong P, Ma X, Nie W, Chen Y, Zhang J, Dong Q, Yang M, Wang W (2020). Mouse umbilical cord mesenchymal stem cell paracrine alleviates renal fibrosis in diabetic nephropathy by reducing myofibroblast transdifferentiation and cell proliferation and upregulating MMPs in mesangial cells. J Diabetes Res.

[46] Li B, Luan S, Chen J, Zhou Y, Wang T, Li Z, Fu Y, Zhai A, Bi C (2020). The MSC-derived exosomal lncRNA H19 promotes wound healing in diabetic foot ulcers by upregulating PTEN via MicroRNA-152-3p. Mol Ther Nucleic Acids.

[47] Ding J, Wang X, Chen B, Zhang J, Xu J (2019). Exosomes derived from human bone marrow mesenchymal stem cells stimulated by deferoxamine accelerate cutaneous wound healing by promoting angiogenesis. Biomed Res Int.

[48] Ti D, Hao H, Tong C, Liu J, Dong L, Zheng J, Zhao Y, Liu H, Fu X, Han W (2015). LPS-preconditioned mesenchymal stromal cells modify macrophage polarization for resolution of chronic inflammation via exosome-shuttled let-7b. J Transl Med.

[49] Yu M, Liu W, Li J, Lu J, Lu H, Jia W, Liu F (2020). Exosomes derived from atorvastatin-pretreated MSC accelerate diabetic wound repair by enhancing angiogenesis via AKT/eNOS pathway. Stem Cell Res Ther.

[50] Ahmed A, Getti G, Boateng J (2018). Ciprofloxacin-loaded calcium alginate wafers prepared by freeze-drying technique for potential healing of chronic diabetic foot ulcers. Drug Deliv Transl Res.

[51] Ahmed EM (2015). Hydrogel: preparation, characterization, and applications: a review. J Adv Res.

[52] Khayambashi P, Iyer J, Pillai S, Upadhyay A, Zhang Y, Tran SD (2021). Hydrogel encapsulation of mesenchymal stem cells and their derived exosomes for tissue engineering. Int J Mol Sci.

[53] Mardpour S, Ghanian MH, Sadeghi-Abandansari H, Mardpour S, Nazari A, Shekari F, Baharvand H (2019). Hydrogel-mediated sustained systemic delivery of mesenchymal stem cell-derived extracellular vesicles improves hepatic regeneration in chronic liver failure. ACS Appl Mater Interfaces.

[54] Catoira MC, Fusaro L, Di Francesco D, Ramella M, Boccafoschi F (2019). Overview of natural hydrogels for regenerative medicine applications. J Mater Sci Mater Med.

[55] Eivazzadeh-Keihan R, Aliabadi HAM, Radinekiyan F, Sobhani M, Farzane K, Maleki A, Madanchi H, Mahdavi M, Shalan AE (2021). Investigation of the biological activity, mechanical properties and wound healing application of a novel scaffold based on lignin-agarose hydrogel and silk fibroin embedded zinc chromite nanoparticles. RSC Adv.

[56] Assi R, Foster TR, He H, Stamati K, Bai H, Huang Y, Hyder F, Rothman D, Shu C, Homer-Vanniasinkam S, Cheema U, Dardik A (2016). Delivery of mesenchymal stem cells in biomimetic engineered scaffolds promotes healing of diabetic ulcers. Regen Med.

[57] Guo J, Hu H, Gorecka J, Bai H, He H, Assi R, Isaji T, Wang T, Setia O, Lopes L, Gu Y, Dardik A (2018). Adipose-derived mesenchymal stem cells accelerate diabetic wound healing in a similar fashion as bone marrow-derived cells. Am J Physiol Cell Physiol.

[58] Dong Y, Rodrigues SAM, Li X, Kwon SH, Kosaric N, Khong S, Gao Y, Wang W, Gurtner GC (2017). Injectable and tunable gelatin hydrogels enhance stem cell retention and improve cutaneous wound healing. Adv Funct Mater.

[59] Dong Y, Rodrigues M, Kwon SH, Li X, Brett SAEA, Elvassore N, Wang W, Gurtner GC (2018). Acceleration of diabetic wound regeneration using an in situ-formed stem-cell-based skin substitute. Adv Healthcare Mater.

[60] Chen S, Shi J, Zhang M, Chen Y, Wang X, Zhang L, Tian Z, Yan Y, Li Q, Zhong W, Xing M, Zhang L, Zhang L (2015). Mesenchymal stem cell-laden anti-inflammatory hydrogel enhances diabetic wound healing. Sci Rep.

[61] Dumortier G, Grossiord JL, Agnely F, Chaumeil JC (2006). A review of poloxamer 407 pharmaceutical and pharmacological characteristics. Pharm Res.

[62] Kaisang L, Siyu W, Lijun F, Daoyan P, Xian CJ, Jie S (2017). Adipose-derived stem cells seeded in Pluronic F-127 hydrogel promotes diabetic wound healing. J Surg Res.

[63] Wu YY, Jiao YP, Xiao LL, Li MM, Liu HW, Li SH, Liao X, Chen YT, Li JX, Zhang Y (2018). Experimental study on effects of adipose-derived stem cell-seeded silk fibroin chitosan film on wound healing of a diabetic rat model. Ann Plast Surg.

[64] Bai H, Kyu-Cheol N, Wang Z, Cui Y, Liu H, Liu H, Feng Y, Zhao Y, Lin Q, Li Z (2020). Regulation of inflammatory microenvironment using a self-healing hydrogel loaded with BM-MSCs for advanced wound healing in rat diabetic foot ulcers. J Tissue Eng.

[65] Ahmed R, Afreen A, Tariq M, Zahid AA, Masoud MS, Ahmed M, Ali I, Akram Z, Hasan A (2020). Bone marrow mesenchymal stem cells preconditioned with nitric oxide releasing chitosan/PVA hydrogel attenuate diabetic wound healing in rabbits. Biomed Mater.

[66] Jin X, Shang Y, Zou Y, Xiao M, Huang H, Zhu S, Liu N, Li J, Wang W, Zhu P (2020). Injectable hypoxia-induced conductive hydrogel to promote diabetic wound healing. ACS Appl Mater Interfaces.

[67] Xu Q, Gao SAY, Guo L, Creagh-Flynn J, Zhou D, Greiser U, Dong Y, Wang F, Tai H, Liu W, Wang W, Wang W (2018). A hybrid injectable hydrogel from hyperbranched PEG macromer as a stem cell delivery and retention platform for diabetic wound healing. Acta Biomater.

[68] Feng J, Mineda K, Wu SH, Mashiko T, Doi K, Kuno S, Kinoshita K, Kanayama K, Asahi R, Sunaga A, Yoshimura K (2017). An injectable non-cross-linked hyaluronic-acid gel containing therapeutic spheroids of human adipose-derived stem cells. Sci Rep.

[69] Li H, Dai Y, Shu J, Yu R, Guo Y, Chen J (2015). Spheroid cultures promote the stemness of corneal stromal cells. Tissue Cell.

[70] da Silva LP, Santos TC, Rodrigues DB, Pirraco RP, Cerqueira MT, Reis RL, Correlo VM, Marques AP (2017). Stem cell-containing hyaluronic acid-based spongy hydrogels for integrated diabetic wound healing. J Invest Dermatol.

[71] Chen Z, Zhang B, Shu J, Wang H, Han Y, Zeng Q, Chen Y, Xi J, Tao R, Pei X, Yue W, Han Y (2020). Human decellularized adipose matrix derived hydrogel assists mesenchymal stem cells delivery and accelerates chronic wound healing. J Biomed Mater Res A.

[72] Srifa W, Kosaric N, Amorin A, Jadi O, Park Y, Mantri S, Camarena J, Gurtner GC, Porteus M (2020). Cas9-AAV6-engineered human mesenchymal stromal cells improved cutaneous wound healing in diabetic mice. Nat Commun.

[73] Aijaz A, Teryek M, Goedken M, Polunas M, Olabisi RM (2019). Coencapsulation of ISCs and MSCs enhances viability and function of both cell types for improved wound healing. Cell Mol Bioeng.

[74] Abbasi N, Hamlet S, Love RM, Nguyen N-T (2020). Porous scaffolds for bone regeneration. J Sci Adv Mater Dev.

[75] Lutzweiler G, Halili AN, Vrana NE (2020). The overview of porous, bioactive scaffolds as instructive biomaterials for tissue regeneration and their clinical translation. Pharmaceutics.

[76] Han F, Dong Y, Su Z, Yin R, Song A, Li S (2014). Preparation, characteristics and assessment of a novel gelatin-chitosan sponge scaffold as skin tissue engineering material. Int J Pharm.

[77] Dhandayuthapani B, Yoshida Y, Maekawa T, Kumar DS (2011). Polymeric scaffolds in tissue engineering application: a review. Int J Polym Sci.

[78] Gunathilake TMSU, Ching YC, Ching KY, Chuah CH, Abdullah LC (2017). Biomedical and microbiological applications of bio-based porous materials: a review. Polymers (Basel).

[79] O'Loughlin A, Kulkarni M, Creane M, Vaughan EE, Mooney E, Shaw G, Murphy M, Dockery P, Pandit A, O'Brien T (2013). Topical administration of allogeneic mesenchymal stromal cells seeded in a collagen scaffold augments wound healing and increases angiogenesis in the diabetic rabbit ulcer. Diabetes.

[80] Tong C, Hao H, Xia L, Liu J, Ti D, Dong L, Hou Q, Song H, Liu H, Zhao Y, Fu X, Han W (2016). Hypoxia pretreatment of bone marrow-derived mesenchymal stem cells seeded in a collagen-chitosan sponge scaffold promotes skin wound healing in diabetic rats with hindlimb ischemia. Wound Repair Regen.

[81] Annaidh AN, Bruyere K, Destrade M, Gilchrist MD, Ottenio M (2012). Characterization of the anisotropic mechanical properties of excised human skin. J Mech Behav Biomed Mater.

[82] Örgül D, Eroğlu H, Tiryaki M, Pınarlı FA, Hekimoglu S (2021). In-vivo evaluation of tissue scaffolds containing simvastatin loaded nanostructured lipid carriers and mesenchymal stem cells in diabetic wound healing. J Drug Deliv Sci Technol.

[83] Chen TY, Wen TK, Dai NT, Hsu SH (2021). Cryogel/hydrogel biomaterials and acupuncture combined to promote diabetic skin wound healing through immunomodulation. Biomaterials.

[84] Mohanty C, Pradhan J (2020). A human epidermal growth factor-curcumin bandage bioconjugate loaded with mesenchymal stem cell for in vivo diabetic wound healing. Mater Sci Eng C Mater Biol Appl.

[85] Yang BY, Hu CH, Huang WC, Ho CY, Yao CH, Huang CH (2019). Effects of bilayer nanofibrous scaffolds containing curcumin/lithospermi radix extract on wound healing in streptozotocin-induced diabetic rats. Polymers (Basel).

[86] Augustine R, Zahid AA, Hasan A, Wang M, Webster TJ (2019). CTGF loaded electrospun dual porous core-shell membrane for diabetic wound healing. Int J Nanomedicine.

[87] Augustine R, Rehman SRU, Ahmed R, Zahid AA, Sharifi M, Falahati M, Hasan A (2020). Electrospun chitosan membranes containing bioactive and therapeutic agents for enhanced wound healing. Int J Biol Macromol.

[88] Eltom A, Zhong G, Muhammad A (2019). Scaffold techniques and designs in tissue engineering functions and purposes: a review. Adv Mater Sci Eng.

[89] Ng R, Zang R, Yang KK, Liu N, Yang S-T (2012). Three-dimensional fibrous scaffolds with microstructures and nanotextures for tissue engineering. RSC Adv.

[90] Jun I, Han HS, Edwards JR, Jeon H (2018). Electrospun fibrous scaffolds for tissue engineering: viewpoints on architecture and fabrication. Int J Mol Sci.

[91] Bruzauskaite I, Bironaite D, Bagdonas E, Bernotiene E (2016). Scaffolds and cells for tissue regeneration: different scaffold pore sizes-different cell effects. Cytotechnology.

[92] Chen S, Wang H, Su Y, John JV, McCarthy A, Wong SL, Xie J (2020). Mesenchymal stem cell-laden, personalized 3D scaffolds with controlled structure and fiber alignment promote diabetic wound healing. Acta Biomater.

[93] Hou C, Shen L, Huang Q, Mi J, Wu Y, Yang M, Zeng W, Li L, Chen W, Zhu C (2013). The effect of heme oxygenase-1 complexed with collagen on MSC performance in the treatment of diabetic ischemic ulcer. Biomaterials.

[94] He S, Shen L, Wu Y, Li L, Chen W, Hou C, Yang M, Zeng W, Zhu C (2015). Effect of brain-derived neurotrophic factor on mesenchymal stem cell-seeded electrospinning biomaterial for treating ischemic diabetic ulcers via milieu-dependent differentiation mechanism. Tissue Eng Part A.

[95] Tam K, Cheyyatraviendran S, Venugopal J, Biswas A, Choolani M, Ramakrishna S, Bongso A, Fong CY (2014). A nanoscaffold impregnated with human wharton's jelly stem cells or its secretions improves healing of wounds. J Cell Biochem.

[96] Navone SE, Pascucci L, Dossena M, Ferri A, Invernici G, Acerbi F, Cristini S, Bedini G, Tosetti V, Ceserani V, Bonomi A, Pessina A, Freddi G, Alessandrino A, Ceccarelli P, Campanella R, Marfia G, Alessandri G, Parati EA (2014). Decellularized silk fibroin scaffold primed with adipose mesenchymal stromal cells improves wound healing in diabetic mice. Stem Cell Res Ther.

[97] Tapias LF, Ott HC (2014). Decellularized scaffolds as a platform for bioengineered organs. Curr Opin Organ Transplant.

[98] Chu J, Shi P, Deng X, Jin Y, Liu H, Chen M, Han X, Liu H (2018). Dynamic multiphoton imaging of acellular dermal matrix scaffolds seeded with mesenchymal stem cells in diabetic wound healing. J Biophotonics.

[99] Beiki B, Zeynali B, Seyedjafari E (2017). Fabrication of a three dimensional spongy scaffold using human Wharton's jelly derived extra cellular matrix for wound healing. Mater Sci Eng C Mater Biol Appl.

[100] Lee YJ, Baek SE, Lee S, Cho YW, Jeong YJ, Kim KJ, Jun YJ, Rhie JW (2019). Wound-healing effect of adipose stem cell-derived extracellular matrix sheet on full-thickness skin defect rat model: Histological and immunohistochemical study. Int Wound J.

[101] Han Y, Tao R, Han Y, Sun T, Chai J, Xu G, Liu J (2014). Microencapsulated VEGF gene-modified umbilical cord mesenchymal stromal cells promote the vascularization of tissue-engineered dermis: an experimental study. Cytotherapy.

[102] Badylak SF (2007). The extracellular matrix as a biologic scaffold material. Biomaterials.

[103] Wilshaw SP, Kearney J, Fisher J, Ingham E (2008). Biocompatibility and potential of acellular human amniotic membrane to support the attachment and proliferation of allogeneic cells. Tissue Eng Part A.

[104] Nie C, Yang D, Morris SF (2009). Local delivery of adipose-derived stem cells via acellular dermal matrix as a scaffold: a new promising strategy to accelerate wound healing. Med Hypotheses.

[105] Kim PJ, Attinger CE, Steinberg JS, Evans KK (2014). Integra(R) bilayer wound matrix application for complex lower extremity soft tissue reconstruction. Surg Technol Int.

[106] AbouIssa A, Mari W, Simman R (2015). Clinical usage of an extracellular, collagen-rich matrix: a case series. Wounds.

[107] Cazzell S, Vayser D, Pham H, Walters J, Reyzelman A, Samsell B, Dorsch K, Moore M (2017). A randomized clinical trial of a human acellular dermal matrix demonstrated superior healing rates for chronic diabetic foot ulcers over conventional care and an active acellular dermal matrix comparator. Wound Repair Regen.

[108] Kavros SJ, Dutra T, Gonzalez-Cruz R, Liden B, Marcus B, McGuire J, Nazario-Guirau L (2014). The use of PriMatrix, a fetal bovine acellular dermal matrix, in healing chronic diabetic foot ulcers: a prospective multicenter study. Adv Skin Wound Care.

[109] Kosaric N, Kiwanuka H, Gurtner GC (2019). Stem cell therapies for wound healing. Expert Opin Biol Ther.

[110] Rennert RC, Sorkin M, Garg RK, Januszyk M, Gurtner GC (2013). Cellular response to a novel fetal acellular collagen matrix: implications for tissue regeneration. Int J Biomater.

[111] Nie C, Zhang G, Yang D, Liu T, Liu D, Xu J, Zhang J (2015). Targeted delivery of adipose-derived stem cells via acellular dermal matrix enhances wound repair in diabetic rats. J Tissue Eng Regen Med.

[112] Fu J, Zhang Y, Chu J, Wang X, Yan W, Zhang Q, Liu H (2019). Reduced graphene oxide incorporated acellular dermal composite scaffold enables efficient local delivery of mesenchymal stem cells for accelerating diabetic wound healing. ACS Biomater Sci Eng.

[113] Han Y, Sun T, Han Y, Lin L, Liu C, Liu J, Yan G, Tao R (2019). Human umbilical cord mesenchymal stem cells implantation accelerates cutaneous wound healing in diabetic rats via the Wnt signaling pathway. Eur J Med Res.

[114] Moon KC, Suh HS, Kim KB, Han SK, Young KW, Lee JW, Kim MH (2019). Potential of allogeneic adipose-derived stem cell-hydrogel complex for treating diabetic foot ulcers. Diabetes.

[115] Zeng X, Tang Y, Hu K, Jiao W, Ying L, Zhu L, Liu J, Xu J (2017). Three-week topical treatment with placenta-derived mesenchymal stem cells hydrogel in a patient with diabetic foot ulcer: a case report. Medicine (Baltimore).

[116] De Francesco F, Graziano A, Trovato L, Ceccarelli G, Romano M, Marcarelli M, Cusella De Angelis GM, Cillo U, Riccio M, Ferraro GA (2017). A regenerative approach with dermal micrografts in the treatment of chronic ulcers. Stem Cell Rev.

[117] Nuschke A (2014). Activity of mesenchymal stem cells in therapies for chronic skin wound healing. Organogenesis.

[118] Chu DT, Phuong TNT, Tien NLB, Tran DK, Thanh VV, Quang TL, Truong DT, Pham VH, Ngoc VTN, Chu-Dinh T, Kushekhar K (2020). An update on the progress of isolation, culture, storage, and clinical application of human bone marrow mesenchymal stem/stromal cells. Int J Mol Sci.

[119] Calcat ICS, Sanz-Nogues C, O'Brien T (2021). When origin matters: properties of mesenchymal stromal cells from different sources for clinical translation in kidney disease. Front Med (Lausanne).

[120] Marino L, Castaldi MA, Rosamilio R, Ragni E, Vitolo R, Fulgione C, Castaldi SG, Serio B, Bianco R, Guida M, Selleri C (2019). Mesenchymal stem cells from the Wharton's Jelly of the human umbilical cord: biological properties and therapeutic potential. Int J Stem Cells.

[121] Garcia-Bernal D, Garcia-Arranz M, Yanez RM, Hervas-Salcedo R, Cortes A, Fernandez-Garcia M, Hernando-Rodriguez M, Quintana-Bustamante O, Bueren JA, Garcia-Olmo D, Moraleda JM, Segovia JC, Zapata AG (2021). The current status of mesenchymal stromal cells: controversies, unresolved issues and some promising solutions to improve their therapeutic efficacy. Front Cell Dev Biol.

[122] Rosemann A, Bortz G, Vasen F, Sleeboom-Faulkner M (2016). Global regulatory developments for clinical stem cell research: diversification and challenges to collaborations. Regen Med.

[123] Maderal AD, Vivas AC, Eaglstein WH, Kirsner RS (2012). The FDA and designing clinical trials for chronic cutaneous ulcers. Semin Cell Dev Biol.

[124] Driver VR, Lavery LA, Reyzelman AM, Dutra TG, Dove CR, Kotsis SV, Kim HM, Chung KC (2015). A clinical trial of integra template for diabetic foot ulcer treatment. Wound Repair Regen.

